# Exploration of Electronic Health Record Patterns of Emergency Physicians—Charting the Digital Burden

**DOI:** 10.1001/jamanetworkopen.2024.29749

**Published:** 2024-08-01

**Authors:** Lisa S. Rotenstein, Melanie Molina

**Affiliations:** Divisions of Clinical Informatics and General Internal Medicine, Department of Medicine, University of California, San Francisco;; Center for Physician Experience and Practice Excellence, Brigham and Women’s Hospital, Boston, Massachusetts;; Department of Emergency Medicine, University of California, San Francisco;; Zuckerberg San Francisco General Hospital, San Francisco, California.

Amidst continued high demand for emergency department (ED) services, staffing shortages, and an ongoing ED capacity crisis across the US, recent surveys have estimated ED physicians to have the highest rates of burnout.^1^ While many factors contribute to physician burnout, time spent on the electronic health record (EHR) is a known factor associated with physicians’ emotional exhaustion.^2^ However, the burden of the EHR for ED physicians has not been well characterized.

In this study, Iscoe et al^3^ seek to develop the evidence base regarding ED physicians’ EHR use, leveraging use data from 3 EDs within a single health care system in the Northeast. Based on 79 physicians who provided more than 35 000 encounters to unique patients, Iscoe et al^3^ found that ED physicians spent a median (IQR) of 6.82 (3.70–11.25) minutes on the EHR per encounter, with most of this time spent on EHR-based documentation. Iscoe et al^3^ additionally identify specific factors (handoffs, patient acuity, discharge against medical advice) associated with greater EHR time expenditure.

The estimates in the study by Iscoe et al^3^ are consistent with prior estimates of ED attending physician EHR time expenditure gathered through direct observation (6.5 minutes per patient encounter).^4^ This correspondence between audit log and observation-based estimates suggests the viability of audit log data for estimating EHR-related work in the ED, despite known limitations of this data source (eg, only captures time actively interacting with the EHR). The estimates of EHR time expenditure described in this study by Iscoe et al^3^ notably fall below those for ED trainees (estimated mean EHR time per patient chart of 29 minutes)^5^ and those of nonprocedural physicians (eg, with primary care specialists spending a mean of 19.8 minutes per encounter).^6^ These differences should be interpreted in the context of differential productivity expectations, with ED attending physicians likely expected to see more patients per clinical session than trainee or outpatient physician colleagues. Nevertheless, the differences in encounter-level time expenditure between ED attending physicians and other groups likely suggest a need for EHR optimization approaches uniquely tailored to the workflows and demands of ED physicians.

The study by Iscoe et al^3^ notably demonstrates that ED physicians spent almost 4 times as much time—a median (IQR) of 3.72 (2.15–5.88) minutes vs 1.05 (0.28–2.45) minutes—on documentation compared with EHR review. While prior studies have characterized documentation as the largest source of EHR time expenditure across specialties,^7^ it is unclear what the right ratio of time is for emergency medicine, where rapid integration of patients’ histories and past presentations is crucial for accurate and quick decision-making. The findings from Iscoe et al^3^ raise the question of whether this level of documentation burden, in addition to contributing to physician burnout, is appropriate for optimal clinical care. Iscoe et al^3^ also underscore the important potential of ambient documentation technologies to alleviate the burden of the EHR for physicians and potentially enable reallocation of time to activities critical for medical decision-making, such as EHR review.

Not surprisingly, the study by Iscoe et al^3^ also found that after adjusting for patient age, severity of presentation, chief complaint, and practice setting, physician handoffs were associated with approximately 33% more time per encounter (2 additional minutes per encounter on a median of 6 minutes). Given the high-risk nature of clinical handoffs, this additional time is likely justified. Prior research has demonstrated the cognitive load costs associated with handoffs, as well as the fact that physicians are more likely to make errors during handoffs in care.^8^ Accordingly, in the future, technologies that can effectively summarize relevant clinical courses and patient information to reduce the time and cognitive burden of the clinical handoff may be particularly valuable in the ED setting.

Finally, Iscoe et al^3^ found that an emergency severity index (ESI) of 1, indicating the highest level of illness acuity, was associated with almost 3 times as many minutes spent in the EHR per encounter compared with the lowest acuity ESI of 5. At first glance, this would seem logical from a clinical perspective, with greater medical decision-making, documentation, and EHR review time needed for higher-acuity encounters. However, recent research has suggested that the assignment of ESI is subject to bias, especially racial-, ethnic-, and language-related biases, with patients identifying as Black, Hispanic, or other race or ethnicity having less acute triage scores than White peers.^9^ Future research should assess the extent to which lesser time spent on lower severity ED cases holds across racial, ethnic, and language groups within ESI groups, and to what extent biased ESI assignment might bias the time physicians devote to individual ED cases with similar clinical presentations.

While the study by Iscoe et al^3^ makes important contributions to benchmarking physicians’ EHR time, its conclusions should be interpreted in the context of several methodologic points. As previously noted, time captured via the user action log (or audit log) is thought to be an underestimate of true time spent on patient encounters. This point is particularly salient in the ED, where physicians may spend time in acute clinical situations or performing procedures without simultaneously interacting with the EHR. Additionally, ED workflows vary across institutions and practice settings, and findings by Iscoe et al^3^ should be interpreted in that context. For example, the study by Iscoe et al^3^ includes encounters in which a resident physician or advanced practice clinician was involved in the encounter, supervised by an attending physician. In these cases, an attending physician may only have to attest a resident or advanced practice clinician’s note, thus reducing the time associated with documentation, and lowering overall study estimates of EHR time. Finally, at present, the EHR use activity log cannot estimate time spent on the ED trackboard, which can be used to track results and active workups across multiple patients, and, according to the prior work by Iscoe et al,^10^ represents the second greatest source of EHR time expenditure at the shift level.

Despite these limitations, the study by Iscoe et al^3^ makes important contributions to characterizing the EHR-based work of ED physicians. This preliminary understanding is critical for optimizing ED physicians’ EHR-based workflows and ensuring high quality care delivery, supported, rather than encumbered, by the EHR.
